# Hardware Implementation of Differential Oscillatory Neural Networks Using VO_ 2_-Based Oscillators and Memristor-Bridge Circuits

**DOI:** 10.3389/fnins.2021.674567

**Published:** 2021-07-16

**Authors:** Jafar Shamsi, María José Avedillo, Bernabé Linares-Barranco, Teresa Serrano-Gotarredona

**Affiliations:** Instituto de Microelectrónica de Sevilla (CSIC), Universidad of Sevilla, Seville, Spain

**Keywords:** oscillatory neural networks, relaxation oscillators, coupled oscillators, vanadium dioxide, memristor, Hopfield Neural Network, associative memory

## Abstract

Oscillatory Neural Networks (ONNs) are currently arousing interest in the research community for their potential to implement very fast, ultra-low-power computing tasks by exploiting specific emerging technologies. From the architectural point of view, ONNs are based on the synchronization of oscillatory neurons in cognitive processing, as occurs in the human brain. As emerging technologies, VO_2_ and memristive devices show promising potential for the efficient implementation of ONNs. Abundant literature is now becoming available pertaining to the study and building of ONNs based on VO_2_ devices and resistive coupling, such as memristors. One drawback of direct resistive coupling is that physical resistances cannot be negative, but from the architectural and computational perspective this would be a powerful advantage when interconnecting weights in ONNs. Here we solve the problem by proposing a hardware implementation technique based on differential oscillatory neurons for ONNs (DONNs) with VO_2_-based oscillators and memristor-bridge circuits. Each differential oscillatory neuron is made of a pair of VO_2_ oscillators operating in anti-phase. This way, the neurons provide a pair of differential output signals in opposite phase. The memristor-bridge circuit is used as an adjustable coupling function that is compatible with differential structures and capable of providing both positive and negative weights. By combining differential oscillatory neurons and memristor-bridge circuits, we propose the hardware implementation of a fully connected differential ONN (DONN) and use it as an associative memory. The standard Hebbian rule is used for training, and the weights are then mapped to the memristor-bridge circuit through a proposed mapping rule. The paper also introduces some functional and hardware specifications to evaluate the design. Evaluation is performed by circuit-level electrical simulations and shows that the retrieval accuracy of the proposed design is comparable to that of classic Hopfield Neural Networks.

## Introduction

Brain efficiency in cognitive processing relies on an architecture made up of distributed processors (neurons) and memories (synapses). Inspired by this brain architecture, novel cognitive processing paradigms are now being developed which go beyond the Von Neumann model. In addition to architecture, the devices employed also play a significant role in power and area efficiency. In this regard, emerging low power and compact devices constitute alternative resources useful for developing efficient cognitive processors.

Synthetic neural networks (NNs) have been under development for more than 50 years. Different types of NNs have been introduced, differentiated by their neuron types and data representation. Examples include classic artificial neural networks (ANNs) (Jain et al., 1996), spiking neural networks (SNNs) (Paugam-Moisy and Bohte, 2012), and oscillatory neural networks (ONNs) (Izhikevich, 1997). In classic ANNs, neurons are the simplest biological computing units that accumulate weighted input values prior to the application of an activation function to obtain the output value. In these neural networks, data representation is based on binary or real numbers. In SNNs, which have more biological features, spiking neurons receive and generate spikes, the spiking time or frequency of which is exploited for processing. In ONNs, an oscillator acts as a neuron, and the oscillator phase is the main characteristic used for processing. Like coupled oscillators, neurons process information by synchronizing. It has been hypothesized that synchronization plays an important role in cognitive processing (Gupta et al., 2016). Similarly to synapses, coupling functions describe the connections between the oscillators (Stankovski et al., 2015). A coupling function determines how one oscillator will affect another.

With regard to hardware, emerging devices such as vanadium dioxide (VO_2_) (Velichko et al., 2017) and memristors (Strukov et al., 2008) make it feasible to implement oscillators and coupling functions efficiently. VO_2_ devices have been exploited to design compact nano-scale, low power oscillators (Parihar et al., 2015; Shukla et al., 2015; Velichko et al., 2017; Raychowdhury et al., 2019; Corti et al., 2020a). A basic VO_2_ oscillator circuit can be made by a series connection of a VO_2_ device and a resistor or a CMOS transistor (Corti et al., 2020b). As coupling components, resistors and/or a capacitors are typically used to interconnect the oscillators. Resistively and capacitively coupled oscillators have shown their potential for processing tasks, such as image recognition (Corti et al., 2018) or vertex coloring (Weiher et al., 2021). For the case of a pair of VO_2_ oscillators, when a pure resistor is employed for coupling them, increasing the connectivity strength (decreasing the resistance) tends to put the oscillator pair in phase, while for capacitively coupling, increasing the coupling capacitance tends to put the oscillator pair in anti-phase (Parihar et al., 2015). Although parallel resistors and capacitors make it easier to form a different phase difference between a coupled oscillator pair, it is not so easy to adjust the connection strength. Moreover, a capacitor usually consumes a large area of the hardware. For implementing adjustable connections, the memristor is a convenient device (Li et al., 2018; Sung et al., 2018; Camuñas-Mesa et al., 2019), as its non-volatile resistance can be adjusted to the desired value. However, the implementation of negative and zero weights is an issue. One proposed method is to use an extra memristor crossbar array to implement negative weights (Alibart et al., 2013; Molahasani Majdabadi et al., 2020). It is also possible to achieve both in phase and in anti-phase oscillations in a pair of coupled VO_2_ oscillators using a single resistor as a coupling component (Corti et al., 2020a). In this method, a specific range of resistances (high resistance) puts the oscillator pair in anti-phase, while low resistances puts it in phase. Therefore, high resistance ranges and low resistance ranges could be equivalent to using negative and positive weights, respectively. However, this has only been studied for small size of ONNs, and scaling up to arbitrary size needs further research.

Here we propose another method, in which oscillatory neurons are made of pairs of VO_2_ oscillators, coupled to be in anti-phase. This way, each neuron provides two differential outputs in anti-phase, and these differential oscillatory neurons are interconnected with adjustable positive- or negative-weight memristor-bridge circuits (Adhikari et al., 2012; Shamsi et al., 2017) to implement differential oscillatory neural networks (DONNs). First, a differential oscillatory neuron based on VO_2_ devices is proposed to benefit from its differential outputs with anti-phase signals. The oscillatory neurons’ differential output allows the memristor-bridge circuit to implement positive, negative, and zero weights. Differential oscillatory neurons are then interconnected through memristor-bridge circuits to form a Hopfield-type architecture (Hopfield, 1982) as an associative memory. The synaptic weights are calculated using the standard Hebbian rule and mapped to the memristors’ resistances in the memristor-bridge circuits. Simulation results demonstrate the pattern retrieval capability of the proposed architecture.

To the best of our knowledge, this is the first attempt to introduce and implement differential oscillatory neural networks (DONNs). The main contribution of this paper is the integration of differential oscillatory neurons and memristor-bridge circuits to implement DONNs. Our study also introduces several criteria which can be used to evaluate and compare different implementations of ONNs.

The rest of the paper is organized as follows. Section “Methods” describes the architecture of DONNs, including a brief introduction to ONNs, VO_2_ devices, and memristors. Section “Differential oscillatory neural networks (DONNs)” looks at circuit designs for DONNs. A differential oscillator neuron is proposed and the memristor-bridge circuit is introduced as an inter-neuron coupling function. The differential oscillatory neurons and memristor-bridge circuits are then combined to implement fully connected DONNs. Section “Evaluation Method” introduces some design specifications as design criteria for evaluating our design, and Section “Results” provides evaluation and simulation results, followed by some conclusions.

## Methods

### Oscillatory Neural Networks

An ONN is a dynamic system that comprises weakly connected oscillatory neurons and is described through Ordinary Differential Equations (ODEs) (Izhikevich, 1997; Nakao, 2016).

(1) $${\dot{x}}_{\text{i}}=f_{\text{i}}\left(x_{\text{i}}\right)+\sum_{j=1}^{n}w_{\text{ij}}g_{\text{ij}}\left(x_{\text{i}},x_{\text{j}}\right),i=1,2,\ldots ,n$$

where *n* is the number of the oscillatory neurons and *x*_i_ is the state vector of oscillatory neuron *i* (***x****_i_* ∈ ℝ^*m*^, *m* ≥ 2), which is a function of its phase^1^. Function *f*_i_(*x*_i_) describes the dynamic behavior of the oscillatory neuron *i* and is usually formulated using *m*-dimensional differential equations. Parameter *w*_ij_ is the weight between oscillatory neurons *i* and *j*. *g*_ij_(*x_i_,x_j_*) is the coupling function that shows the effect of oscillatory neuron *j* on oscillatory neuron *i.* The weights, *w*_ij_, and the coupling function, *g*_ij_(*x_i_,x_j_*), are crucial factors in oscillatory neural networks’ behavior.

From the hardware perspective, oscillator function *f*_i_(*x*_i_) can be implemented using both harmonic and relaxation oscillators. In this paper, a relaxation oscillator based on VO_2_ devices is used. On the other hand, different coupling functions have been introduced in literature, such as a sinus function in the Kuramoto model (Acebrón et al., 2005) and a phase detector in PLL-based oscillators (Hoppensteadt and Izhikevich, 2000). One of the most straightforward coupling functions in terms of implementation is diffusive coupling, where *g*_ij_(*x_i_,x_j_*) = (*x_j_-x_i_*) (Stankovski et al., 2015). The diffusive coupling function is implemented by connecting the oscillatory neuron outputs via resistors (memristors), creating what is known as resistively coupled oscillatory neurons (Corti et al., 2020a). Moreover, using a mapping method, the weights *w*_ij_ are mapped to resistance values *R*_ij_. Using VO_2_-based oscillatory neurons and memristive devices, all elements of an oscillatory neural network can therefore be implemented. The following subsections summarize the main features and models of VO_2_ and memristor devices.

#### Vanadium Dioxide (VO_2_) Device Model

A VO_2_ device^2^ is a two-terminal device based on a phase change material that presents insulator-to-metal (IMT) and metal-to-insulator transitions (MIT) (Velichko et al., 2017). The transition is temperature-driven, caused by the joule heating in the presence of an applied voltage. Increasing the device temperature causes a change from a high resistance (*R*_*H*_) state to a low resistance (*R*_*L*_) state and vice versa.

Although the temperature of the device is the main factor that causes the transitions, device behavior is also shaped by the applied voltage. In (Maffezzoni et al., 2015), a SPICE model for VO_2_ devices is introduced where the transitions are related to a high voltage *V*_*H*_ and a low voltage threshold *V*_*L*_. When an increasing applied voltage reaches *V*_*H*_, the resistance changes from its high resistance value *R*_*H*_ = 1/*G*_*L*_ to its low resistance value *R*_*L*_ = 1/*G*_*H*_. When a decreasing applied voltage drops below *V*_*L*_, a transition from the low resistance state to the high resistance state occurs. The time constant of the transitions is *τ*. This model is compatible with fabricated VO_2_ devices, making it a reliable model for SPICE simulations. Although it is a compact SPICE model, it makes use of a discontinuous nonlinear function, which may yield convergence problems when simulating large scale circuits. To prevent this problem, we introduce here an equivalent mathematical macro model that uses a continuously differentiable nonlinear function, as follows:

(2) $$\dot{g}=\frac{K}{\tau }\left[-g+\left(\frac{G_{H}+G_{L}}{2}\right)+\left(\frac{G_{H}-G_{L}}{2}\right)\times S\left(v,g\right)\right]$$

where,

(3) $$S\left(v,g\right)=tanh\left(A.\left(V_{H}-V_{L}\right).\left(\frac{v-V_{L}}{V_{H}-V_{L}}-\frac{g-G_{L}}{G_{H}-G_{L}}\right)\right)$$

**FIGURE 1:**
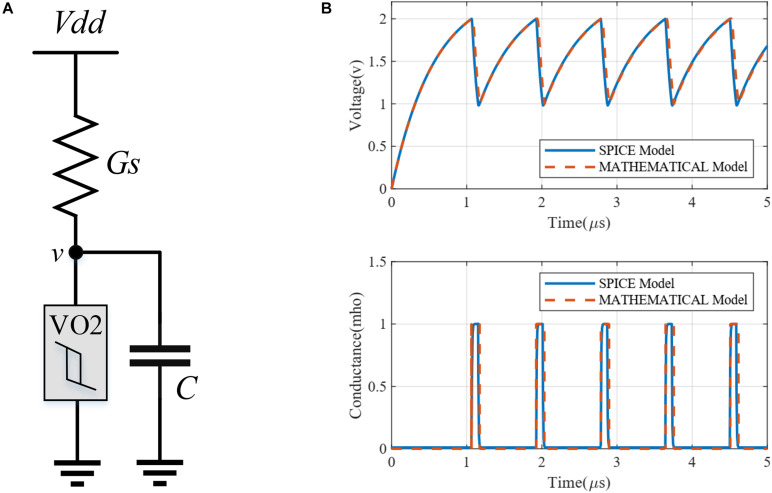
**(A)** A basic oscillator circuit. **(B)** Simulation of the basic oscillator with Maffezzoni’s SPICE model and the proposed mathematical model. The simulation parameters are *V*_*H*_ = 2, *V*_*L*_ = 1, *R*_*L*_ = 1 kΩ, *R*_*H*_ = 100 kΩ, *τ* = 30 ns, and *K* = 6.

Variables *g* and *v* are the conductance and voltage of the VO_2_ device, respectively. Parameter *A* is a large constant value, and the other parameters are the main parameters of a VO_2_ device. Parameter *K* is a fitting parameter to match this mathematical model with the original SPICE model. A basic circuit of a VO_2_-based oscillator was simulated to compare the proposed mathematical model with the Maffezzoni SPICE model (see Figure 1A). The differential equation of the circuit is given by:

(4) $$\begin{array}{c} \dot{v}=\frac{1}{C}.\left(-\left(G_{s}+g\right)v+G_{s}.Vdd\right)\\ \dot{g}=G\left(g,v\right) \end{array}$$

where G(g,v) is given by the right-hand expression in Eq. (2). Figure 1B shows the simulation results of the proposed mathematical model matching very well with Maffezzoni’s SPICE model.

#### Memristor Model

A memristor is a two-terminal resistive device whose resistance is adjustable. It is typically used as an analog memory that can be both non-volatile or volatile (Ohno et al., 2011; van den Hurk et al., 2014; La Barbera et al., 2015; Wang et al., 2017; Ascoli et al., 2021). In addition, the adjustability of non-volatile memristors and their nanoscale size make them attractive candidates to implement massive adjustable synaptic circuits, especially with crossbar structures.

In some models, the memristor behavior is described by using one positive and one negative threshold voltages (−| *V*_*n*_| and *V*_*p*_) (Ascoli et al., 2013; Krestinskaya et al., 2020). In memristors with counterclockwise switching, when the applied memristor voltage is larger than the positive threshold voltage *V*_*p*_, the resistance of the memristor decreases. On the contrary, in memristors with clockwise switching, applying a voltage larger than *V*_*p*_ causes an increase in resistance, while the resistance decreases when the applied voltage is less than −| *V*_*n*_| (Min and Cho, 2021). With an applied voltage between −| *V*_*n*_| and *V*_*p*_, the resistance remains constant.

The operational phases of a memristor are usually known as programming and operating phases (Shamsi et al., 2018). In the programming phase, the amplitude of each positive (negative) voltage pulse, applied across the memristor, is set to some pre-defined value larger than the modulus of the memristor threshold voltage *V*_*p*_ (| *V*_*n*_ |). Depending on the number, polarity, height and width of the pulses, the resistance is adjusted. On the other hand, in the operating phase, the maximum and minimum peaks of the pulses are within the intervals between the upper and lower threshold voltages, so there is no resistance change. This paper focuses on the operating phase of the memristor as a non-volatile synaptic circuit. We thus consider a memristor as a constant resistor, and weights are mapped to the resistance values. Consequently, throughout this paper we consider that memristor terminal voltages never exceed the threshold voltages.

Resistance range and threshold voltages are closely related to the materials used in memristor fabrication (Hadiyawarman et al., 2018), which therefore constitute important factors for memristor-based circuit designs. In this paper, the threshold voltages and the resistance ranges are assumed to meet the needs of our design. Specifically, we assume that maximum voltages across the memristors are never larger than the high threshold voltage *V*_*H*_ of the VO_2_ devices. Also, some relations for defining a valid range of resistances are introduced in subsection “Circuit Design Method.”

### Differential Oscillatory Neural Networks (DONNs)

**FIGURE 2:**
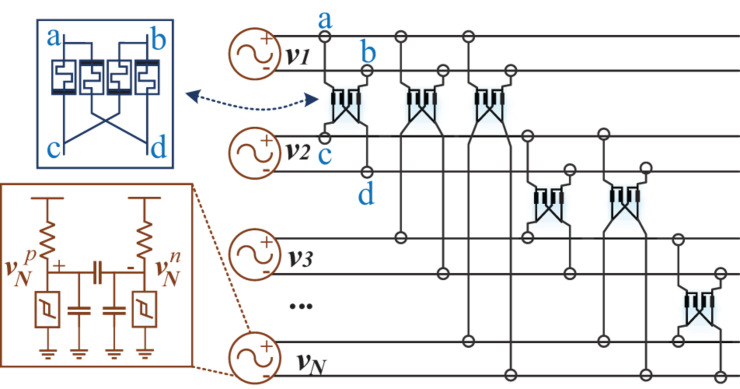
Hardware architecture of the fully connected DONN. Differential neurons are implemented with a pair of VO_2_ oscillators, oscillating in anti-phase. Synapses are implemented using memristor-bridge circuits.

In this study, differential oscillatory neurons and memristor-bridge circuits are combined to implement a fully connected DONN, similar to a classic (non-oscillatory) Hopfield neural network. The proposed neural network circuit, shown in Figure 2, is used as an associative memory. The following subsections describe the differential oscillatory neurons and memristor-bridge circuits used as the basic blocks of the DONN.

#### Differential Oscillatory Neuron Circuit

**FIGURE 3:**
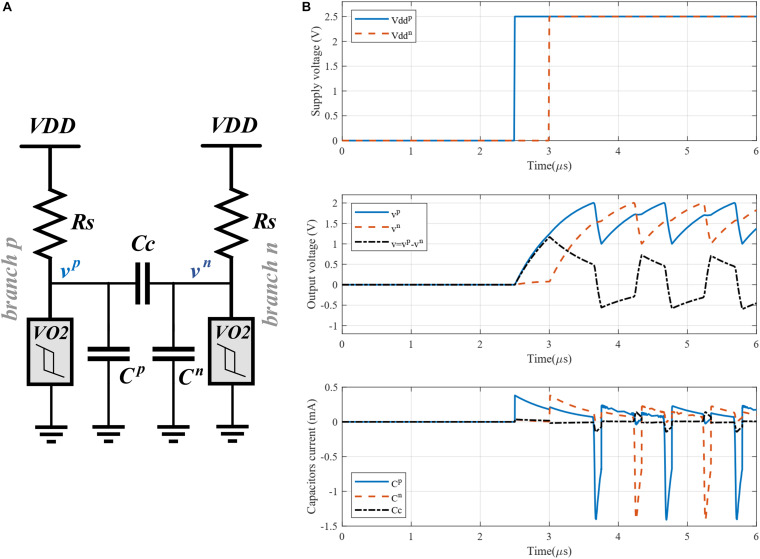
**(A)** Proposed differential oscillatory neuron (*C*^*p*^ = *C*^*n*^ = *C*). **(B)** Simulation of the differential oscillatory neuron with power-on delay initialization; The positive and negative voltage sources were applied at *t* = 2.5 μs and *t* = 3 μs, respectively. Bottom figure shows the current through the capacitors.

Figure 3A shows the circuit of the proposed differential oscillatory neuron which generates two anti-phase signals. It comprises two single-ended oscillators connected to each other through a capacitor. The coupling capacitor forces the single-ended oscillators to be in anti-phase, in which they produce differential signals. To get anti-phase waveforms, the coupling capacitor *Cc* should be much lower than *C* (*Cc* < < *C*) (Parihar et al., 2015). However, when *Cc* > > *C* the outputs tend to be in phase. In addition, when *Cc* = *C* the output signals do not increase monotonically causing distorted output signals (Parihar et al., 2015). The following equations describe the dynamics of the differential oscillatory neuron.

(5) $$\begin{array}{c} {\dot{v}}^{p}=\frac{1}{C+C_{C}}\left[-\left(G_{s}+g^{p}\right)v^{p}+C_{C}.{\dot{v}}^{n}+G_{s}.Vdd\right]\\ {\dot{v}}^{n}=\frac{1}{C+C_{C}}\left[-\left(G_{s}+g^{n}\right)v^{n}+C_{C}.{\dot{v}}^{p}+G_{s}.Vdd\right]\\ {\dot{g}}^{p}=G\left(g^{p},v^{p}\right)\\ {\dot{g}}^{n}=G\left(g^{n},v^{n}\right) \end{array}$$

where indexes *p* and *n* indicate the corresponding branch *p* and *n*, respectively.

The starting point for exploiting a DONN is the initialization phase, in which an input pattern is applied to the DONN. The power-on delay method can be used to initialize the differential oscillatory neuron (Corti et al., 2020b). In this method, the power supplies of the single-ended oscillator branches are applied in a power-on sequence with a specific delay. Given an input value, the power supply is applied to one of the branches first, and then, after a given time, to the second branch. For binary values, the delay time should be half of the oscillators’ period. For instance, when the binary value is 1, the power supply is applied to the positive branch first, and then, after one half period, to the negative branch. Figure 3B shows the simulation results of a differential oscillatory neuron for which the period is 1 μs and the delay between the applied power supplies is 0.5 μs. The output voltages are anti-phase shown in the middle of Figure 3B.

In our design, parameters *C*, *C_*C*_, R_*s*_*, and *Vdd* were calculated using relations that satisfy the operating conditions of the DONN circuit (see subsection “Circuit Design Method”). Some relations are necessary to ensure the *VO*_2_ devices are biased in their negative resistance region to guarantee the respective branches perform as oscillators. Using these relations, parameters *R*_*s*_ and *Vdd* are calculated. Other relations are used to calculate parameters *Cc* and *C*. Details of the operating conditions are provided in subsection “Circuit Design Method.”

#### Synaptic Memristive-Bridge Circuit

**FIGURE 4:**
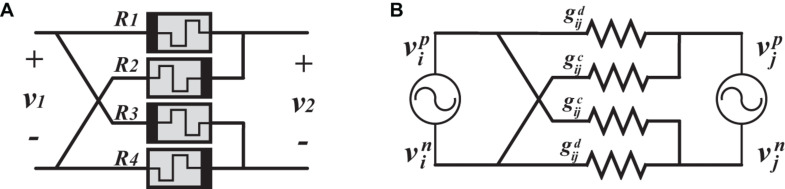
**(A)** Memristor-bridge circuit. **(B)** Equivalent circuit of two differential oscillatory neurons coupled through one memristor-bridge circuit.

As an analogy of the Wheatstone bridge, the memristor-bridge is introduced to implement a synaptic circuit capable of providing positive, negative, and zero weights (Figure 4A; Adhikari et al., 2012). Being a four-terminal circuit makes it appropriate for differential structures. However, it is also used in single-ended neural networks (Jackson et al., 2016; Shamsi et al., 2017). Figure 4B shows the equivalent circuit with two differential oscillatory neurons coupled by the memristor-bridge circuit. The following conditions are required to implement a symmetric coupling function.

(6) $$g_{\text{ij}}^{\text{d}}=\frac{1}{R_{1}}=\frac{1}{R_{4}}$$

(7) $$g_{\text{ij}}^{\text{c}}=\frac{1}{R_{2}}=\frac{1}{R_{3}}$$

where superscript *d* stands for “direct” path (positive with positive or negative with negative), while superscript *c* stands for “crossed” path (positive with negative, and vice versa). Conductance $g_{\text{ij}}^{\text{d}}$ is located between the positive branches (negative branches), tending to put positive branches (negative branches) in phase. When the positive branches (negative branches) are in phase, then the two differential oscillatory neurons are considered in phase. On the other hand, conductance $g_{\text{ij}}^{\text{c}}$ is located between a positive and a negative branch, thus tending to put them in phase while the positive branches (negative branches) are anti-phase. When the positive branches (negative branches) are anti-phase, then the two differential oscillators are considered as anti-phase. Considering the values of $g_{\text{ij}}^{\text{d}}$ and $g_{\text{ij}}^{\text{c}}$, differential oscillatory neurons tend to be in phase (anti-phase) when $g_{\text{ij}}^{\text{d}}>g_{\text{ij}}^{\text{c}}$ ($g_{\text{ij}}^{\text{d}}<g_{\text{ij}}^{\text{c}}$). In this regard, it is possible to implement a coupling circuit with a positive (if $g_{\text{ij}}^{\text{d}}>g_{\text{ij}}^{\text{c}}$), negative (if $g_{\text{ij}}^{\text{d}}<g_{\text{ij}}^{\text{c}}$), or zero (if $g_{\text{ij}}^{\text{d}}=g_{\text{ij}}^{\text{c}}$) weight.

It also worth mentioning that the maximum voltage across a memristor is kept less than *V*_*H*_. Suppose that a memristor is connected to the output nodes of two *VO*_2_ devices from two distinct differential oscillatory neurons. Therefore, the maximum voltage across the memristor will be | *V_*H*_−V_*L*_*|. However, during the initialization *Vdd* is applied with a delay to a branch that may increase the voltage of one terminal of the memristor up to *V*_*H*_, while the voltage of the other terminal is around zero. Thus, the maximum voltage across a memristor will in general be less than *V*_*H*_.

The following subsection reviews the standard Hebbian rule used to calculate the synaptic weights. A mapping method is also introduced to map the synaptic weights to the conductance values $g_{\text{ij}}^{\text{d}}$ and $g_{\text{ij}}^{\text{c}}$.

##### Training and mapping rules

A training rule is used to store patterns in neural networks, adjusting the synaptic weights accordingly. Once the weights are known, we propose a mapping rule to obtain the physical resistances for the memristor-bridge synapses. To store patterns in the DONN, we use the Hebbian rule to calculate the weights.

(8) $$w_{\text{ij}}=\frac{1}{L}\sum_{k=1}^{P}b_{\text{i}}^{k}b_{\text{j}}^{k}\;i,j\in \left\{1,2,3,\ldots ,L\right\}$$

where *P* is the number of stored patterns and *L* is the number of pixels in each pattern (which is equal to the number of neurons in the DONN). Elements *b*_i_ and *b*_j_ of all stored patterns are used to calculate the weight *w*_ij_.

We propose here the following rules to map the sign and value of the above weights to the memristors’ resistances. Weights *w*_ij_ are mapped to the *g*_ij_ values using the following relation.

(9) $$Map_{value}:g_{\text{ij}}=\{\begin{array}{c} \frac{g_{0}}{1+\beta \times P\times {\left|\frac{1}{w_{\text{ij}}}\right|}_{norm}},w_{\text{ij}}\neq 0\\ \frac{g_{0}}{1+\beta \times P}\;,w_{\text{ij}}=0 \end{array}$$

where *g*_0_ is the maximum conductance (inverse the minimum resistance) of the memristors. Parameter β is a small positive value (e.g., 0.2) that controls the mapping range for conductance *g*_ij_. A larger value for β provides a larger range for conductance *g*_ij_. Value |1/*w*_ij_|_*norm*_ is the Min-Max normalization of |1/*w*_ij_|. In order to obtain the Min-Max normalization, the following relation is used.

(10) $${\left|\frac{1}{w_{\text{ij}}}\right|}_{norm}=\frac{\left|\frac{1}{wij}\right|-min}{max-min}$$

where *min* and *max* are the minimum and maximum values of |1/*w*_ij_| among all non-zero weights, respectively. The following mapping rule was also used to map the weight signs to resistance values $g_{\text{ij}}^{\text{d}}$ and $g_{\text{ij}}^{\text{c}}$.

(11) $$Map_{sign}:\{\begin{array}{c} \alpha g_{\text{ij}}^{\text{d}}=g_{\text{ij}}^{\text{c}}=g_{\mathrm{ij},}w_{\text{ij}}<0\\ \begin{array}{c} g_{\text{ij}}^{\text{d}}=\alpha g_{\text{ij}}^{\text{c}}=g_{\mathrm{ij},}w_{\text{ij}}>0\\ \alpha g_{\text{ij}}^{\text{d}}=\alpha g_{\text{ij}}^{\text{c}}=g_{\mathrm{ij},}w_{\text{ij}}=0 \end{array} \end{array}$$

where α > 1 is a constant value.

The design parameters of the memristor-bridge are *w*_ij_, α, β, and *g*_0_. Parameter *g*_0_ will be obtained based on correctly functioning hardware, as explained next in subsection “Circuit Design Method.” Section “Results” then illustrates how the DONN performance depends on parameters α and β.

#### Circuit Design Method

**FIGURE 5:**
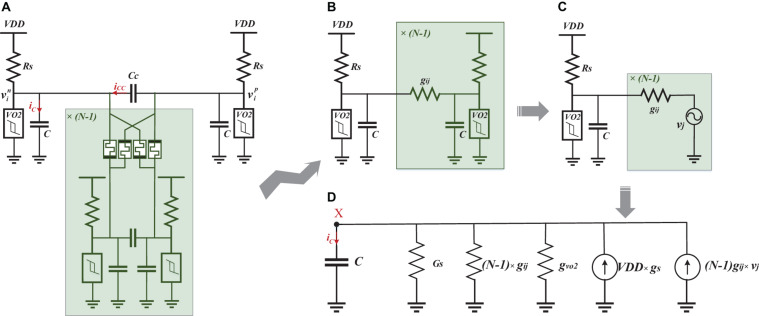
Simplification of the DONN. **(A)** The DONN from the viewpoint of one of the differential oscillatory neurons connected to the other *(N−1)* ones. **(B)** The circuit as simplified by ignoring the current *i*_*CC*_, and assuming that the oscillatory neurons are either in phase or in anti-phase. **(C)** Replacement of the *(N−1)* oscillatory neurons with voltage sources. **(D)** Norton equivalent of the simplified circuit.

As a starting point, design relations are extracted to calculate circuit parameters by analyzing the fully connected DONN circuit (Figure 2). Figure 5A shows the circuit from the viewpoint of a single differential oscillatory neuron coupled to other *(N−1)* differential oscillatory neurons. The dynamics of the former differential oscillatory neuron is described by:

(12) $$\begin{array}{c} {\dot{v}}_{\text{i}}^{p}=\frac{1}{C+C_{C}}\left[-\left(G_{s}+g_{\text{i}}^{p}\right)v_{\text{i}}^{p}+C_{C}.{\dot{v}}_{\text{i}}^{n}+G_{s}.Vdd\right]+\sum_{j=1}^{N}i_{\text{ij}}^{p}\\ {\dot{v}}_{\text{i}}^{n}=\frac{1}{C+C_{C}}\left[-\left(G_{s}+g_{\text{i}}^{n}\right)v_{\text{i}}^{n}+C_{C}.{\dot{v}}_{\text{i}}^{p}+G_{s}.Vdd\right]+\sum_{j=1}^{N}i_{\text{ij}}^{n}\\ {\dot{g}}_{\text{i}}^{p}=G\left(g_{\text{i}}^{p},v_{\text{i}}^{p}\right)\\ {\dot{g}}_{\text{i}}^{n}=G\left(g_{\text{i}}^{n},v_{\text{i}}^{n}\right) \end{array}$$

Where $i_{\text{ij}}^{p}$ and $i_{\text{ij}}^{n}$ are the currents from oscillatory neuron *j* to the positive and negative branch of oscillatory neuron *i*, respectively:

(13) $$\begin{array}{c} i_{\text{ij}}^{p}=g_{\text{ij}}^{\text{d}}\left(v_{\text{j}}^{p}-v_{\text{i}}^{p}\right)+g_{\text{ij}}^{\text{c}}\left(v_{\text{j}}^{n}-v_{\text{i}}^{p}\right)\\ i_{\text{ij}}^{n}=g_{\text{ij}}^{\text{c}}\left(v_{\text{j}}^{p}-v_{\text{i}}^{n}\right)+g_{\text{ij}}^{\text{d}}\left(v_{\text{j}}^{n}-v_{\text{i}}^{n}\right) \end{array}$$

In order to guarantee that each VO_2_ device branch operates as an oscillator, the following condition is imposed:

• Each VO_2_ device operates in its negative resistance region: *V*_Max_ > *V*_H_ and *V*_*min*_ < *V*_*L*_, where *V*_*Max*_ (*V*_*min*_) is the maximum (minimum) output voltage if IMT (MIT) does not occur in the VO_2_ device.

This condition guarantees that the voltage of the oscillators do not reach stable points, resulting in permanent oscillation (Raychowdhury et al., 2019). The DONN circuit is analyzed next to extract relations for *V*_*Max*_ and *V*_*min*_. For the analysis, and to simplify the circuit, some assumptions are considered. First, we consider a single point for analysis where the voltage of the positive branch of a differential oscillatory neuron is either at its maximum or minimum value. Regarding the assumption *C* >> *C*_*C*_, the share of current *i*_*CC*_ in the total current *i*_*C*_ is considered negligible. For instance, the current of capacitor *C* (in positive and negative branches) and *C*_*C*_ are shown at the bottom of Figure 3B. When the voltage of the positive branch is at its maximum value, immediately before the jump, the current through capacitors *C*^*p*^ and *C*_*C*_ are 71 and 7.5 μA, respectively. Also, when the voltage of positive branch is at its minimum value, immediately before the jump, the current through capacitors *C*^*p*^ and *C*_*C*_ are 686 and 76 μA, respectively. Thus we omit capacitor *Cc*. It is also assumed that the differential oscillatory neurons with respect to each other are either in phase or in anti-phase. When two differential oscillatory neurons are in phase, the voltage of their positive branches (negative branches) is equal. Therefore, the current through the memristors between the positive branches (negative branches) is zero. Similarly, when in anti-phase, the current through the memristors between a positive and a negative branch is zero, as well. In this regard, the circuit is simplified, as shown in Figure 5B. The simplified circuit is a single-ended oscillator connected to the other *(N−1)* single-ended oscillators. We are also able to replace the *(N−1)* single-ended oscillators with *(N−1)* voltage sources, which generate the same signals than the respective single-ended oscillators (Figure 5C). The circuit is now simple enough to be analyzed easily. The Norton equivalent circuit is shown in Figure 5D. We use next some worst-case operating conditions to extract design relations. In other words, relations are extracted for the most difficult operating conditions to ensure that the calculated parameters are valid for all conditions.

Consequently, parameters *G*_*H*_, *G*_*L*_, *V*_*H*_, and *V*_*L*_ are used in the analysis so that the worst-case conditions are satisfied. In this regard, the maximum (minimum) voltage at node *X* in Figure 5D is obtained for the charging (discharging) period. Then, parameters *G*_*H*_, *G*_*L*_, *V*_*H*_, and *V*_*L*_ are used to find the minimum (maximum) voltage of the charged (discharged) capacitor after the charging (discharging) period, which should be larger (smaller) than *V*_*H*_ (*V*_*L*_). It worth mentioning that IMT (MIT) in VO_2_ devices occurs before reaching the maximum (minimum) voltage of the capacitor/VO_2_ devices, consequently causing permanent oscillation.

(14) $$V_{Max}^{p}=\frac{G_{s}V_{dd}+g\left(N-1\right)V_{L}}{\left(G_{s}+G_{L}+\left(N-1\right)g\right)}>V_{H}$$

(15) $$V_{min}^{p}=\frac{G_{s}V_{dd}+g\left(N-1\right)V_{H}}{\left(G_{s}+G_{H}+\left(N-1\right)g\right)}<V_{L}$$

These relations are used to calculate the values for *G*_*S*_ and *g*_0_. In this regard, we first use relations with *N* = 1 to find a range for *Gs* and a value in this range is selected for *Gs*. Afterward, using the same equations with the selected value of *Gs* and the number of differential oscillatory neurons *N*, a range for *g* is obtained. The maximum value of this range is used as *g*_0_. Finally, we substitute all parameters in the equations to verify whether the selected values are valid. Otherwise, the same procedure is repeated with a new value for *Gs*.

The values for *C* and *C*_*C*_ are other design parameters that need to be considered. It is worth mentioning that a large coupling capacitance value provides strong coupling. On the other hand, a large value for the *Cc/C* ratio causes degradation of the waveform shapes, resulting in deviations from ideal anti-phase waveforms for differential oscillatory neurons (Parihar et al., 2015). Strong coupling therefore has to be traded off against ideal anti-phase waveforms. In this paper, the following relation between *C* and *C*_*C*_ is used.

(16) $$C_{c}\approx 0.1\times C$$

Also, the oscillator period relation is used to calculate *C* and *C*_*C*_ (Corti et al., 2020a).

(17) $$\begin{array}{c} T=C^{*}\times [\left(\frac{1}{G_{L}+G_{s}}\right)ln\left(\frac{V_{max}-V_{L}}{V_{max}-V_{H}}\right)\\ \;+\left(\frac{1}{G_{H}+G_{s}}\right)ln\left(\frac{V_{min}-V_{L}}{V_{min}-V_{H}}\right)] \end{array}$$

where $V_{max}=\frac{G_{s}Vdd}{G_{L}+G_{s}}$ and $V_{min}=\frac{G_{s}Vdd}{G_{H}+G_{s}}$. Parameter *C*^∗^ is the capacitance at the output nodes of the differential oscillatory neurons and is approximately considered as *C*^∗^ ≈ *C* + *C*_*c*_. In the relations mentioned earlier, the input parameters were *Vdd* (voltage supply of the circuit), *V*_*H*_ (high threshold voltage of the VO_2_ device), *V*_*L*_ (low threshold voltage of the VO_2_ device), *G*_*H*_ (high conductance value of the VO_2_ device in the metallic state), *G*_*L*_ (low conductance value of the VO_2_ device in the insulator state), and *N* (the number of neurons in the neural network).

### Evaluation Method

**FIGURE 6:**
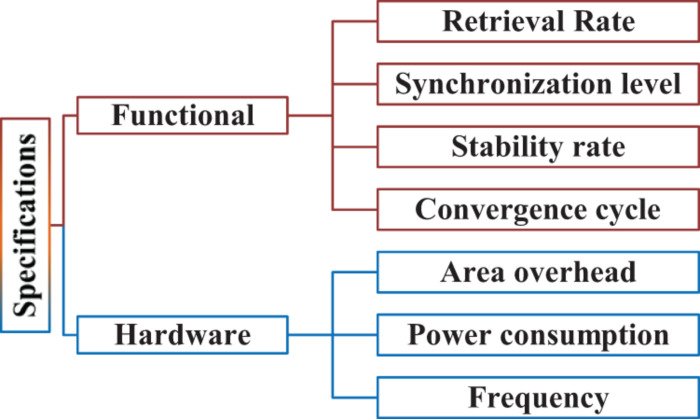
Functional and hardware specifications for evaluating the design.

Figure 6 shows the specifications used to evaluate our design. These specifications are classified into two categories: functional and hardware specifications. The functional specifications are defined as follows:

• *Retrieval rate* is defined as a value in the range of [0 1] that shows the ratio of correct retrieved patterns to all applied patterns:

(18) $$RET=\frac{\#correctretrivedpatterns}{\#allpatterns}$$

• *Synchronization level* is defined to measure how many differential oscillatory neurons (neurons^3^) are synchronized either in phase or in anti-phase. For an input pattern *p*, the synchronization level is a value in the range of [0 1] that is related to the average deviations between synchronized signals in a period *c*:

(19) $$SYN_{p}\left(c\right)=<\frac{1}{N}\sum_{i=1}^{N}m\left(PT_{\text{i}}\left(c\right)-PT_{ref}\left(c\right)\right)>$$

**FIGURE 7:**
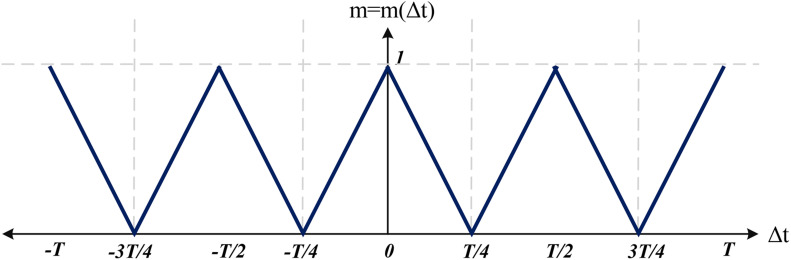
Function *m* = *m(*Δ*t)* used to map a time difference to a value between 0 and 1.

The *SYN_*p*_(c)* value shows the synchronization level at cycle *c*, which is related to the time difference between the peak time of signal *i* at cycle *c*, *PT*_i_(*c*), and the peak time of the reference signal *PT*_*ref*_(*c*) (the signal of positive branches $v_{\text{i}}^{p}$ are used for calculations). Function *m* = *m(*Δ*t)* maps a time difference Δ*t* to a value between 0 to 1 (see Figure 7). It is worth mentioning that time difference Δ*t* is equivalent to phase difference. According to function *m()*, synchronization level *SYN_*p*_(c)* is maximum when the neurons are either in phase (Δ*t* = *0* or Δ*t* = ± *T*) or in anti-phase (Δ*t* = ± *T*/2). When the neurons are not exactly in phase or in anti-phase, a value less than 1 is assigned depending on the phase difference. For instance, the worst case in terms of synchronization corresponds to the phase difference *T*/4.

• *Stability rate* is defined as a value in the range of [0 1] which is the number of applied patterns resulting in stable outputs divided by all applied patterns:

(20) $$STB=\frac{\#patternsresultinginastableoutput}{\#allpatterns}$$

Output stability for an applied pattern is defined based on the average *SYN_*p*_(c)* for all signals in the last few cycles. When this average value is larger than 0.9, the output is considered as a stable output.

• *Convergence cycle* is defined as the number of cycles *Ψ* required to converge to a pattern:

(21) CON=Ψ

where,

(22) $$SYN_{p}\left(\Psi \right)>0.9\times Max_{\forall c>\Psi }\left\{SYN_{p}\left(c\right)\right\}$$

When the output converges to a pattern, the synchronization level is larger than 90% of the maximum synchronization level in subsequent cycles.

In addition to the functional specifications, some hardware specifications are also considered to evaluate the design, such as power consumption per neuron, frequency, and area overhead.

## Results

This section describes the evaluation of the design. A DONN with 15 neurons is first simulated to show how DONNs work^4^. A comprehensive evaluation is then provided to show the performance of DONNs.

### Operation of a DONN

A DONN with *N* = 15 neurons (differential oscillatory neurons) was designed to illustrate the concepts underlying the functional specifications and operation of the DONN.

**FIGURE 8:**
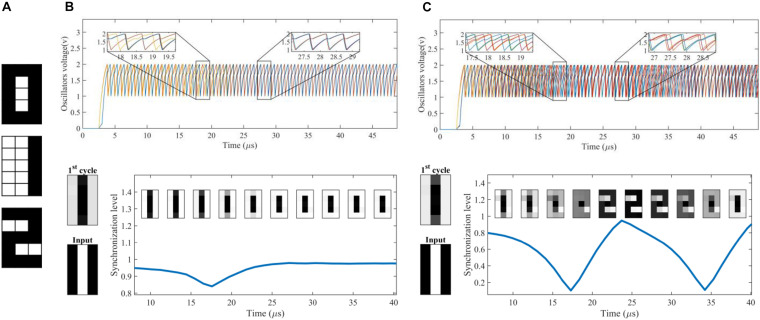
Simulation results of a DONN with 15 neurons. **(A)** Three patterns were stored in the DONN. **(B)** Simulation results for an input pattern with α = 1.8. The top figure shows the voltage of the positive branches of the differential oscillatory neurons. The bottom figure shows the input pattern, the 1st cycle pattern, the synchronization level and the evolution of the retrieved pattern. According to the definition of convergence cycle in Section “Evaluation method,” the ONN requires15 cycles to retrieve the stored pattern. **(C)** A simulation result with a large value for mapping parameter α (α = 10^6^), showing unstable DONN behavior. Although the output converged to a stored pattern, it periodically changed from one pattern to another.

The design relations Eqs. (14)-(17) and the input parameters (the number of neurons *N*, oscillation frequency *f*, supply voltage *Vdd*, and VO_2_ parameters) were used to calculate the circuit parameters. First, Eqs. (14)-(15) were used to calculate values for *g*_0_ and *Gs*. The values for *C* and *C*_*C*_ were then computed using relations Eqs. (16)-(17). After that, the synaptic weights were calculated with the standard Hebbian rule to store the three patterns shown in Figure 8A. Each pattern has 15 pixels and each pixel value at any time is represented by the phase of one of the 15 neurons (each made of two physical VO2 oscillators in anti-phase) with respect to the reference neuron. The rules in Eqs. (9) and (11) were then used to map the weights to the physical synaptic resistance with α = 1.8 and β = 0.2. A summary of the parameters for this design is shown in Table 1.

**TABLE 1:** Design parameters.

Parameter		Value
Number of neurons	*N*	15
Frequency	*f = 1/T*	1 MHz
Supply voltage	*Vdd*	2.5 V
VO_2_ parameters	*V*_*H*_	2 V
	*V*_*L*_	1 V
	*R_*H*_ = 1/G_*L*_*	100 kΩ
	*R_*L*_ = 1/G_*H*_*	1 kΩ
Series resistance	*Rs = 1/Gs*	6 kΩ
Minimum memristor resistance	*r*_0_ = 1/*g*_0_	221 kΩ
Mapping parameters	α	1.8
	β	0.2
Coupling capacitor	*C*_*C*_	11 pF
Parallel capacitor	*C*	108 pF

Figure 8B shows simulation results for an applied input pattern. The voltage of the positive branches can be seen in the top sub-figure. Input patterns are applied by setting a specific initial phase for each neuron. The initial phases of the neurons were set by powering up first the positive branch and then the negative branch of each neuron associated to the white pixels. In contrast, for black pixels, the positive branch was powered up after the negative branch. On the other hand, in order to extract the value of pixel *i* (or neuron *i*) for a specific cycle, the time difference between the voltage peak of the positive branch in neuron *i* with respect to a reference voltage peak (e.g., the voltage of the positive branch in neuron *1*) is measured. For each neuron, if the time difference is zero (half of the period), then a white (black) pixel value is assigned. In Figure 8B (bottom figure) the 1st cycle pattern (which is not among the stored patterns in Figure 8A) is shown. It can be seen that it resembles the input pattern. The same figure also shows the synchronization level during the convergence period. The minimum synchronization level corresponded to a moment in which the deviation between the signals was maximum. The maximum synchronization level occurred when the neurons were either in phase or in anti-phase. The convergence cycle for this pattern was 15 cycles.

Figure 8C shows another simulation of unstable neural network behavior in which the mapping parameter α was a large value (α = 10^6^). For this value, $g_{\text{ij}}^{\text{c}}$ ($g_{\text{ij}}^{\text{d}}$) was a negligible value for positive (negative) weights (see the mapping rule). For zero weights, $g_{\text{ij}}^{\text{c}}$ and $g_{\text{ij}}^{\text{d}}$ were negligible values in comparison to *g*_*0*_ and memristors can be considered as open circuits. The simulation results show that the output was unstable for the input pattern (Figure 8C). The synchronization level changed periodically, and the evolution of the pattern shows that the retrieved pattern periodically changed from one pattern (pattern 0) to another (pattern 2).

### Performance of DONNs

**FIGURE 9:**
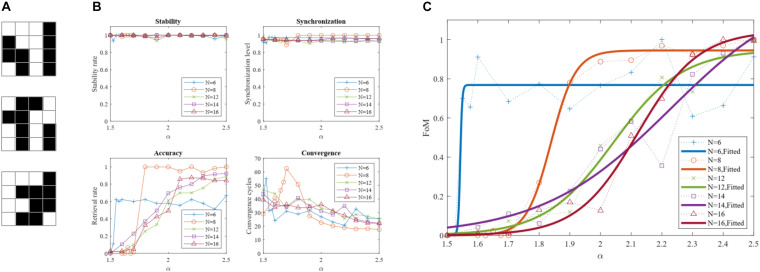
Dependence of the DONN performance on mapping parameter α. **(A)** Three sample patterns generated randomly for a DONN with *N* = 16 neurons. **(B)** DONN specifications versus parameter α. **(C)** Dependence of FoM on mapping parameter α. The dotted lines are the simulation results for the different number of neurons. The solid fitted curves show how the FoM value changed with the value of parameter α.

**FIGURE 10:**
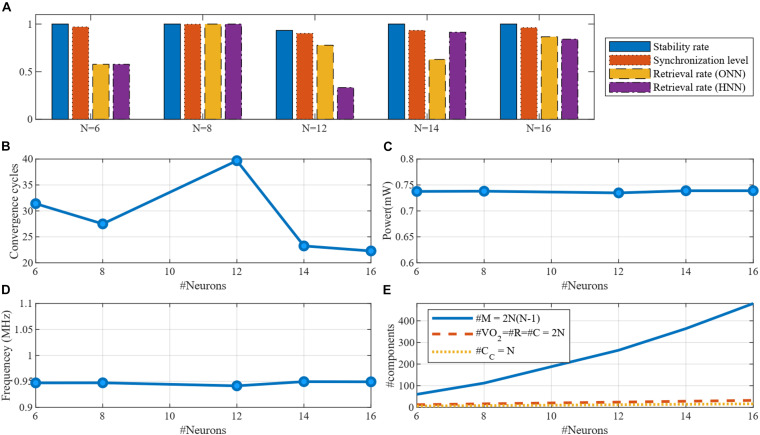
Simulation results of DONNs with different number of neurons. **(A)** Functional specifications of the design including the stability rate, synchronization level, and retrieval rate for both DONN and HNN. **(B)** Average of the convergence cycles for a pattern retrieval. **(C)** Power consumption per differential oscillatory neuron, which was constant with the number of neurons. **(D)** Frequency of the oscillatory neurons. **(E)** Number of components required to implement a fully connected DONN.

The DONN was evaluated with different numbers of neurons *N* using random orthogonal or semi-orthogonal patterns. The hamming distance between orthogonal patterns is exactly half the length of the patterns (number of neurons), while, for semi-orthogonal patterns it is close to half of the length of the patterns but different. Figure 9A shows three samples for 4×4 randomly generated patterns. The ratio of black and white pixels in each pattern was 50%. Approximately 10% of noise was added to the patterns to generate the test patterns. The number of test patterns was 1.5*^*N*^*. The applied noise changed the color of a noisy pixel from black to white or from white to black. The values for the frequency, supply voltage, and VO_2_ device parameters are taken from Table 1. The circuit parameters were calculated in the same way as in the previous case study, and mapping parameter α was selected based on the following Figure-of-Merit for the neural network performance.

(23) $$\begin{array}{c} FoM=<RET>\times <SYN>\times <STB>\\ \;\times <1-|CON{|}_{norm}> \end{array}$$

where |*CON*|_*norm*_ is Min-Max normalization of convergence cycles *CON*, which is calculated using a relation similar to Eq. (10). Parameter *SYN* is the average of *SYN_*p*_(c)* for all signals of all applied patterns in the last few cycles. Figure 9B shows the DONN specifications for different values of α, which were used to calculate the FoM shown in Figure 9C. The simulation was carried out for DONNs with different numbers of neurons *N*, and each point in the figure represents the average of 5 separate simulation results. The number of stored patterns for *N* = 6, 8 was two, while three patterns were stored for the DONNs with *N* = 12, 14, 16. The results show that the performance of the neural network was related to the mapping value α. When value α was 1, performance was zero because all weights were zero (considering Eq. 11, *g*_ij_^d^ = *g*_ij_^c^ for all signs), and the neurons did not affect each other. In this case, the neuron outputs did not change, the output pattern was the same as the input pattern, and the retrieval rate was consequently zero. By increasing the value of parameter α, positive and negative weights were formed, and performance increased. For larger values, however, the network was unstable for some patterns (Figure 8C), and its performance decreased accordingly. With a given performance value, a proper value for parameter α could be selected using Figure 9C. The results of the evaluation of the simulations are shown in Figure 10. Functional specifications versus the number of neurons are shown in Figure 10A. The retrieval rate of the proposed design was compared with the classic HNN, showing comparable accuracy. The stability and synchronization levels show that the output was stable, and that the neurons were synchronized properly. Figure 10B represents the convergence cycles, i.e., the average number of cycles required for the retrieval of patterns. Figure 10C shows power consumption per neuron, which remains constant with the number of neurons, and was about 735 μW or, equivalently, 0.77 pJ/cycle. The frequency of around 950 kHz shown in Figure 10D was around the expected value (1 MHz). The number of components, as an indication of area overhead is given in Figure 10E. The number of memristors scales quadratically with the number of neurons.

(24) #M=2N(N-1)

However, the number of the other components scales linearly with the number of neurons.

Table 2 provides a comparison between the proposed memristive DONN and classic non-oscillatory HNNs. The metrics in the table directly affect the main characteristics of the system which are throughput (convergence time), energy efficiency (power consumption per neuron), and area overhead (hardware complexity). Frequency is applicable for oscillatory neural networks and digital systems, which is 1MHz in the proposed DONN and 500 MHz in the digital hardware introduced in Cai et al. (2020). As a metric for throughput, convergence time is the required time to retrieve a pattern after applying an input. However, this convergence time is highly related to the pattern size (number of neurons in the neural network). The convergence time reported in Cai et al. (2020) is 6.6 μs where the network consists of 60 neurons. In (Hu et al., 2015), it takes 35 μs to retrieve a pattern in an HNN with 3 neurons. In the proposed DONN, the average convergence time is 28.6 cycles, being directly related to the frequency of the neurons. Here, in our simulations, it takes 28.6 μs on average to retrieve a pattern with the frequency being 1 MHz. By increasing the frequency, retrieval time is decreased. For instance, if the frequency would be 500 MHz, the convergence time would be 28.6/500 = 0.027 μs. In the proposed DONN, the power consumption per neuron is 735 μW. One way to improve power consumption is decreasing the voltage of the power supply *V*_*dd*_ and *V*_*H*_, which is possible by reducing the size of the *VO*_2_ devices (Corti et al., 2021). Hardware complexity is another metric to show area overhead, which is provided by counting the number of components. In (Hu et al., 2015), the minimum number of memristors was used, which is half of our design. However, considering other components, DONNs require only basic components (two VO_2_ devices, two resistors, and three capacitors per neuron) which make them potentially compact blocks. On the other hand, classic non-oscillatory HNNs use larger blocks such as amplifiers (8 transistors) and buffers (4 transistors).

**TABLE 2:** Comparison of memristor-based DONNs with non-oscillatory HNNs.

Metrics	The proposed memristive DONN	Mixed-signal Memristive HNN (Sequential operation) (Cai et al., 2020)	Analog Memristive HNN (Hu et al., 2015)
Frequency	1 MHz (oscillators frequency)	500 MHz (clock for the digital part)	N.A (pure analog HNN)
Convergence time (Number of neurons)	28.6 cycles: 28.6 μs with 1 MHz (*N* = 6-16)	6.6 μs (*N* = 60)	35 μs (*N* = 3)
Power consumption per neuron	735 μW (Vdd = 2.5 v)	182 μW	80000 μW
circuit complexity (N is the number neurons)	#Memristor = 2N(N−1) #Capacitor = 3N #Resistor = 2N #VO2 = 2N	# Memristor = N^2^ #Register = N bit #Analog buffer = N #Multiplexer = N bit #Amplifier = 1 #Comparator = 2 #D-FF = 2 #Pass transistor = 2	#Memristor = N(N−1) #Resistor = 5N #Amplifier = N # Analog buffer = N #Inverter = N

One of the main challenges in using emerging devices is cycle-to-cycle and device-to-device variability. Process variations are caused by the immature fabrication technology of these devices (Niu et al., 2010; Maffezzoni et al., 2015; Chaudhuri and Chakrabarty, 2018; Zhu et al., 2020). Although an accurate control of the fabrication process reduces variability, unavoidable variations degrade the performance. Considering Eq. (1), the behavior of oscillators relies on the coupling function *w_ij_.g_ij_(x_i_,x_j_)*, as well as the oscillator function *f_i_(x_i_).* Therefore, the effect of mismatches can be considered for coupling circuits and differential oscillatory neuron circuits, separately. In this regard, we have done preliminary statistical simulations including perturbations in the memristance values and the VO_2_ device parameters. Simulation results show that the FoM is larger than 90% when the mismatch relative sigma *σ* of the memristance is less than 20%. However, the operation of the DONN fails when *σ* ≥ 20%. On the other hand, mismatches in the threshold voltages of the VO_2_ devices (*V_*H*_ and V_*L*_*) are much more critical. In order to have a proper operation of DONNs, absolute sigma should be in the range of 5-10 mV (relative sigma *σ <* 0.5%). Consequently, device manufacturers should be aware that when VO_2_ devices are massively fabricated on a chip, mismatches should have such low sigmas for the threshold voltages.

## Conclusion

A fully connected DONN hardware implementation is proposed comprising VO_2_-based differential oscillatory neurons and memristor-bridge circuits. From the architectural viewpoint, it is like a counterpart of the traditional non-oscillatory Hopfield neural network, used as an associative memory and, as such, the standard Hebbian rule is used to train it. With regard to hardware, two emerging devices are used for the hardware implementation. VO_2_ devices are used to implement the differential oscillatory neurons, and a memristor-bridge circuit with adjustable resistance is used to implement the coupling functions. Finally, the design of the hardware neural network is explained and evaluated using criteria that show the proper operation of the resulting DONNs in terms of synchronization, stability, and pattern retrieval.

## Data Availability Statement

The original contributions presented in the study are included in the article/Supplementary Material, further inquiries can be directed to the corresponding author/s.

## Author Contributions

BL-B and TS-G conceived the initial circuit, which was further developed by JS and MA. JS performed all simulations and circuit analyses. BL-B, MA, and TS-G supervised the work jointly. JS wrote the initial draft of the manuscript which was supervised by all co-authors. All authors contributed to the article and approved the submitted version.

## Conflict of Interest

The authors declare that the research was conducted in the absence of any commercial or financial relationships that could be construed as a potential conflict of interest.

## Footnotes

1 Note that this mathematical phase-based representation method results in an overall dynamical description, which ressembles that of traditional non-oscillatory ANNs.

2 Although VO_2_ devices are negative differential resistance (NDR) devices, they can also be considered as volatile memristors capable of producing oscillatory circuits (Yi et al., 2018), as done throughout the present paper.

3 In the following, the word “neuron” is used for “differential oscillatory neurons.”

4 A presentation with a general overview of DONN is provided in the Supplementary Material section.
